# Inhibition of bcl-2 and cox-2 Protein Expression after Local Application of a New Carmustine-Loaded Clinoptilolite-Based Delivery System in a Chemically Induced Skin Cancer Model in Mice

**DOI:** 10.3390/molecules22112014

**Published:** 2017-11-20

**Authors:** Cristina Mihaela Ghiciuc, Aurel Lulu Strat, Lacramioara Ochiuz, Catalina Elena Lupusoru, Maria Ignat, Aurelia Vasile, Alexandru Grigorovici, Iulian Stoleriu, Carmen Solcan

**Affiliations:** 1 Department of Pharmacology, Faculty of Medicine, “Grigore T. Popa” University of Medicine and Pharmacy, 16, University Street, 700115 Iasi, Romania; cristina.ghiciuc@umfiasi.ro (C.M.G.); alstrat2@yahoo.com (A.L.S.); celupusoru@yahoo.com (C.E.L.); 2 Laboratory of Microbiology, Hospital of Infectious Diseases “Saint Parascheva”, 2, Octav Botez Street, 700116 Iasi, Romania; 3 Department of Pharmaceutical Technology, Faculty of Pharmacy, “Grigore T. Popa” University of Medicine and Pharmacy, 16, University Street, 700115 Iasi, Romania; 4 Faculty of Chemistry, “Al. I. Cuza” University, 11, Blvd. Carol the 1st, 700560 Iasi, Romania; mary_rud@yahoo.com (M.I.); aurelia@uaic.ro (A.V.); 5 Department of Surgery, Faculty of Medicine, “Grigore T. Popa” University of Medicine and Pharmacy, 16, University Street, 700115 Iasi, Romania; alexandrugrigorovici@yahoo.com; 6 Faculty of Mathematics, “Al. I. Cuza” University, 11, Blvd. Carol the 1st, 700506 Iasi, Romania; stoleriu@yahoo.com; 7 Department of Molecular Biology, Histology and Embriology, Faculty of Veterinary Medicine, University of Agricultural Science and Veterinary Medicine “Ion Ionescu de la Brad”, 8, Mihail Sadoveanu Alley, 700489 Iasi, Romania; carmensolcan@yahoo.com

**Keywords:** clinoptilolite, zeolite, drug delivery system, skin cream, Carmustine, chemically induced skin cancer, mouse cancer model, cyclooxygenase 2 (cox-2) protein, B-cell lymphoma-2 (bcl-2) protein, quantitative immunohistochemistry, computer aided diagnostic software

## Abstract

Our research has focused on in vitro and in vivo evaluations of a new Carmustine (BCNU)-loaded clinoptilolite-based delivery system. Two clinoptilolite ionic forms—hydrogen form (HCLI) and sodium form (NaCLI)—were prepared, allowing a loading degree of about 5–6 mg BCNU/g of zeolite matrix due to the dual porous feature of clinoptilolite. Clinoptilolite-based delivery systems released 35.23% of the load in 12 h for the BCNU@HCLI system and only 10.82% for the BCNU@NaCLI system. The BCNU@HCLI system was chosen to develop gel and cream semisolid dosage forms. The cream (C_BCNU@HCLI) released 29.6% of the loaded BCNU after 12 h in the Nylon synthetic membrane test and 31.6% in the collagen membrane test, higher by comparison to the gel. The new cream was evaluated in vivo in a chemically induced model of skin cancer in mice. Quantitative immunohistochemistry analysis showed stronger inhibition of B-cell lymphoma-2 (bcl-2) and cyclooxygenase 2 (cox-2) protein expression, known markers for cancer survival and aggressiveness, after the treatment with C_BCNU@HCLI by comparison to all the control treatment types, including an off-label magistral formula commercially available Carmustine cream as reference, bringing evidence that a clinoptilolite-based delivery systems could be used as a cancer drug carriers and controlled release systems (skin-targeted topical delivery systems).

## 1. Introduction

Skin-targeted topical delivery systems are needed to assure sustained release of the drugs and to maintain a localized effect. New drug delivery systems for local release of antineoplastic agents represent an alternative strategy to systemic administration, having many advantages such as high stability, better bioavailability, and reduced toxic side effects [1,2].

Today, both synthetic and natural zeolites are increasingly used as drug delivery systems for their high loading capacity and controlled release of small drug molecules [3,4,5]. Clinoptilolite is a microporous mineral component of a volcanic rock from the group of natural zeolites that has been extensively tested as a chelating agent or as an adjuvant in cancer therapy, because of its cation exchanging and chelating properties [6,7]. Previously published studies on clinoptilolite confirmed the bimodal porous feature of this material [8,9,10].

**Figure 1:**
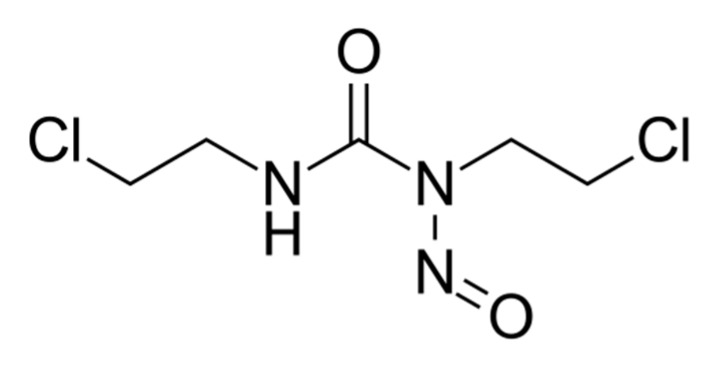
Carmustine (BCNU) chemical formula.

Carmustine (BCNU), a nitrosourea derivative (Figure 1) from the group of cell-cycle phase nonspecific alkylating antineoplastic agents, is used in the treatment of brain tumours and various other malignant neoplasms. It is preferred for the topical administration in early therapy of *Mycosis fungoides*, a haematological type of cancer [11,12].

Potentially, new delivery systems of known therapeutic cancer agents could be evaluated using in vitro and/or in vivo models. Experimental animal models remain essential to discover and test improved delivery methods and systems. A well-known and widely used in vivo model is chemically induced skin cancer in mice, a multistage carcinogenesis model, in which the irreversible initiation occurs following 7,12-dimethylbenz[*a*]anthracene (DMBA) application, and the promotion of the tumour is done with repeated application of 12-*O*-tetradecanoylphorbol-13-acetate (TPA) [13]. 

B-cell lymphoma-2 (bcl-2) protein and cyclooxygenase 2 (cox-2) protein are known tumour markers, expressed in a variety of tumour cells, including skin cancers [13,14], and can be used for the evaluation of the stages of tumour tissue [14,15]. The bcl-2 family of proteins plays a key role as a regulator of epidermal homeostasis and in determining whether a cell will live or die in the case of stressed skin. Deregulation of bcl-2 family members is also involved in skin carcinogenesis [16,17]. Bcl-2, the first protein that was associated with translocations identified in follicular lymphoma, contributes to malignancy by inhibiting apoptosis [18]. Cox-2, the inducible form of this enzyme, contributes to the aggressiveness of the tumour, playing an important role in inflammation and in all steps in the development of solid epithelial tumours (tumour growth, promotion of angiogenesis and inhibition of apoptosis) [15]. In human skin cancer tumours and in skin cancer mouse models, cox-2 levels are frequently correlated with increased skin hyperplasia and decreased apoptosis, leading to tumour development and/or burden [19,20].

Quantitative Immunohistochemistry (QIHC) is an important tool for scientific research in biomarkers detection field. In the last two decades, there has been an increase in publications using QIHC because this technique progressively repositioned itself in relation to other quantitative methods of target detection. In order to make immunohistochemical analysis more objective, quantitative immunohistochemistry analysis techniques based on digital image processing have been developed [21,22] and used successfully in a variety of scientific studies [23,24,25,26,27,28]. Digital values provide a numerical value for each individual case which can resolve the disagreement among different pathologists, whose diagnosis and semi-quantitative evaluation was based on the visual score quantifications. Commercially available programs are limited to color thresholding and pixel counting, approaches that can only provide semi-quantitative assessments as to the amount of chromogen present, reviewed in [21].

This research focuses on clinoptilolite used to prepare a new drug delivery system. The aim of our study was to investigate the qualities of the new delivery system comparatively to a commercially formulation in use, after local application on the tumour, using as control parameters the expression level of bcl-2 and cox-2 proteins.

## 2. Results

### 2.1. Preparation, Porous Parameters and Entrapment Efficacy of the Modified Delivery Clinoptilolite-Based System

**Figure 2:**
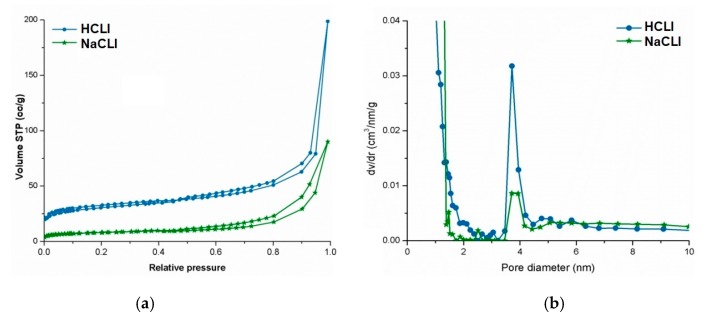
Clinoptilolite matrices: (**a**) N_2_-sorption isotherms; (**b**) pore size distribution curves. Clinoptilolite hydrogen form (HCLI), clinoptilolite sodium form (NaCLI).

N_2_-sorption analysis was used to assess the porous features of the hosts. As observed in Figure 2a, clinoptilolite, in both cationic forms (clinoptilolite hydrogen form abbreviated HCLI and clinoptilolite sodium form abbreviated NaCLI, respectively), show a type I isotherm, according to IUPAC classification [29]. The isotherms rise sharply at the beginning, at low relative pressures (P/P_0_ < 0.5), indicating the adsorption of the N_2_ molecules in the micropores of the adsorbent samples. Moreover, the existence of the second type of porosity within the structure of clinoptilolite was indicated by the hysteresis loop of type H3 according to the International Union of Pure and Applied Chemistry (IUPAC) classification, observed for the N_2_-sorption isotherms recorded for both HCLI and NaCLI samples. The primary clinoptilolite porosity (or microporosity) was caused by its specific inbuilt crystalline structure (channels), but the secondary one came from the sizes of the zeolite and other mineral grains in the rock as well as by structural features of the rock proper [29]. 

The pore size distribution curves (PSD) were an indicative of the pore size homogeneity and were used for calculation of the pore sizes. The PSD curves recorded for HCLI and NaCLI samples, shown in Figure 2b, reflected only the homogeneity and the sizes of the mesopores, since the estimation of PSD from N_2_-sorption data using the classical capillary condensation–evaporation model proposed by Barrett, Joyner, and Halenda (BJH) can be applied only for materials with isolated mesopores and not for the materials with strong connectivity effect (i.e., characterized by the complex pore structure composed of connected pores of various sizes).

The calculated porous features, including specific surface area, pore volume, and the pore diameter are summarized below. The measurements were made in triplicate. Typical surface areas and pore volumes were obtained. As expected, the surface area and the pore volume of the NaCLI sample were lower than for HCLI matrix. This was mainly due to the presence of bulkier Na^+^ cations within the microporous channels of clinoptilolite. Also, the loading degree of both matrices was about 5–6 mg BCNU/g of matrix, the larger amount of BCNU uptake by HCLI comparatively with NaCLI was due to the higher free pore volumes available for absorption. These results are calculated and summarized in Table 1.

It is well known that the CLI framework exhibits three sets of crossing channels that are located in the (010) plane. Two sets of the channels are parallel to the *c*-axis: the A channels are formed by strongly compressed ten-membered rings (aperture of 3.0 × 7.6 Å) and B channels are confined by eight-membered rings (aperture of 3.3 × 4.6 Å). C channels are parallel to the *a*-axis or [102] and are also formed by eight-membered rings (aperture of 2.6 × 4.7 Å) [30]. The size of these channels limits the size of the molecules that can pass through them. Therefore, clinoptilolite is able to act as a molecular sieve. Because carmustine is a small-molecular chemotherapeutic agent (its molecular size resulted from the predicted spherical van der Waals volume being of 4.8 Å [31]), its molecule size is compatible with the A channels aperture of the clinoptilolite framework, formed by 10-membered rings (3.0 × 7.6 Å).

It is found that the degree of the loaded carmustine on both cationic forms of clinoptilolite is different. This result could be explained by the volume, position and intensity of the electrostatic field induced by the Na^+^ and H^+^ compensation cations, respectively.

#### 2.1.1. In Vitro Drug Release Profile and Kinetics of BCNU from Modified Delivery Clinoptilolite-Based Systems

**Figure 3:**
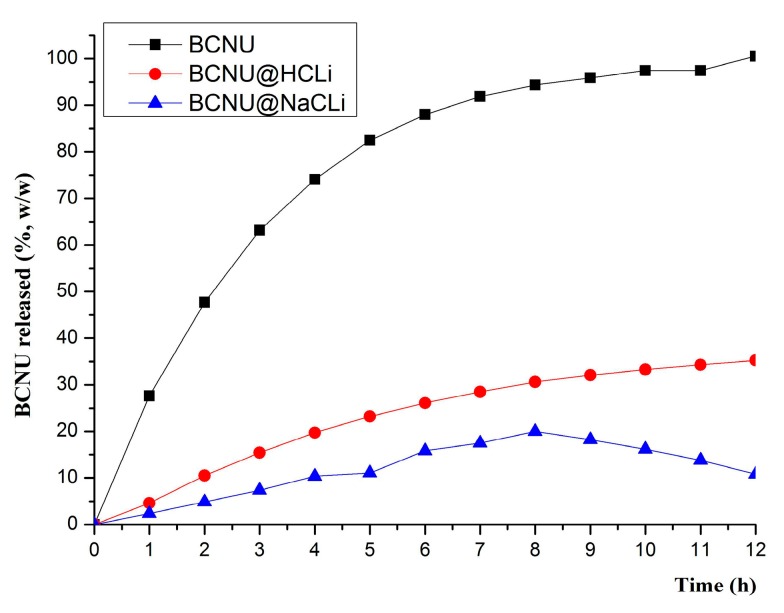
In vitro dissolution release of BCNU from modified delivery clinoptilolite-based systems.

In vitro dissolution tests revealed a prolonged release of BCNU from the two modified delivery clinoptilolite-based system (MDCS) in comparison to the free BCNU dissolution profile (Figure 3).

The modified delivery clinoptilolite-based systems based on monocationic clinoptilolite behaved differently: BCNU@HCLI system released 35.23% of the loaded BCNU in 12 h while BCNU@NaCLI system only 10.82%. The data from the curve fitting analysis of the results obtained from the dissolution test of BCNU from clinoptilolite-based systems, based on the four mathematical models, specific for the dosage forms with modified release (zero-order kinetics, first-order kinetics, Huguchi and Korsmeyer-Peppas models), revealed that the release of BCNU from BCNU@HCLI system is a Fick type diffusion as a consequence of Korsmeyer-Peppas model of data fitting, while the release of BCNU from BCNU@NaCLI system is a first order kinetics mechanism (Table 2).

#### 2.1.2. In Vitro Dissolution BCNU Release Study and Measurement of Permeation Coefficient of BCNU

**Figure 4:**
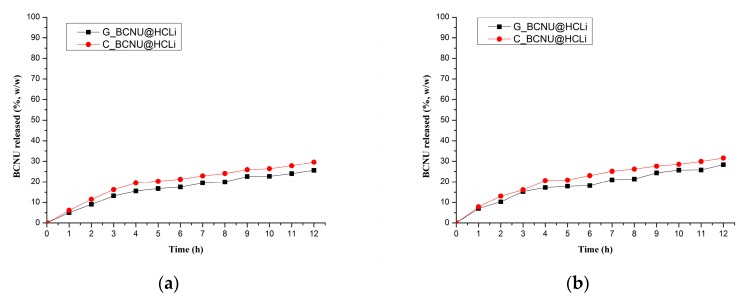
In vitro cumulative release of BCNU from BCNU@HCLI formulated as two types of semisolid dosage forms: (**a**) Nylon membrane; (**b**) Collagen membrane.

In order to emphasize the relevance of the in vitro dissolution test, we have used two types of synthetic membranes: Nylon and a collagen membrane, supplied by Millipore (Merck, Darmstadt, Germany). As can be easily noticed from Figure 4, there are no significant differences among the two types of membranes.

The best releasing characteristics were obtained for the cream C_BCNU@HCLI formulation which released 29.6% of the loaded BCNU after 12 h in the Nylon synthetic membrane test, compared to 31.6% in the collagen membrane test. Both results are higher in comparison to the percentages obtained for the gel formulation. These same types of results are also found in the permeation coefficient values shown in Table 3. 

Based upon these results, the cream formulation, C_BCNU@HCLI, was selected for further testing in vivo in order to evaluate its effects.

### 2.2. Inhibition of bcl-2 and cox-2 Protein Expression after Local Application of a New Modified Delivery Clinoptilolite-Based System Loaded with Carmustine

#### The Chemically Induced Skin Tumour Model in Mice

**Figure 5:**
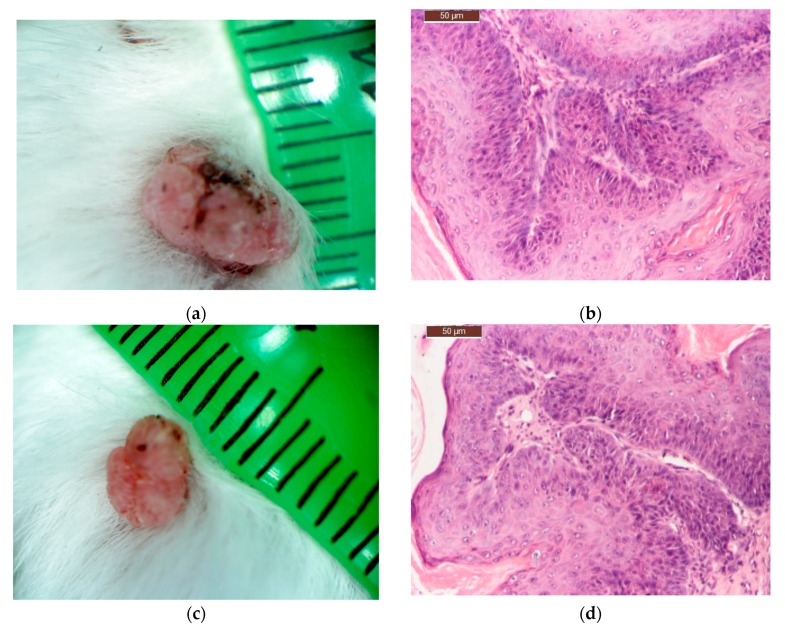

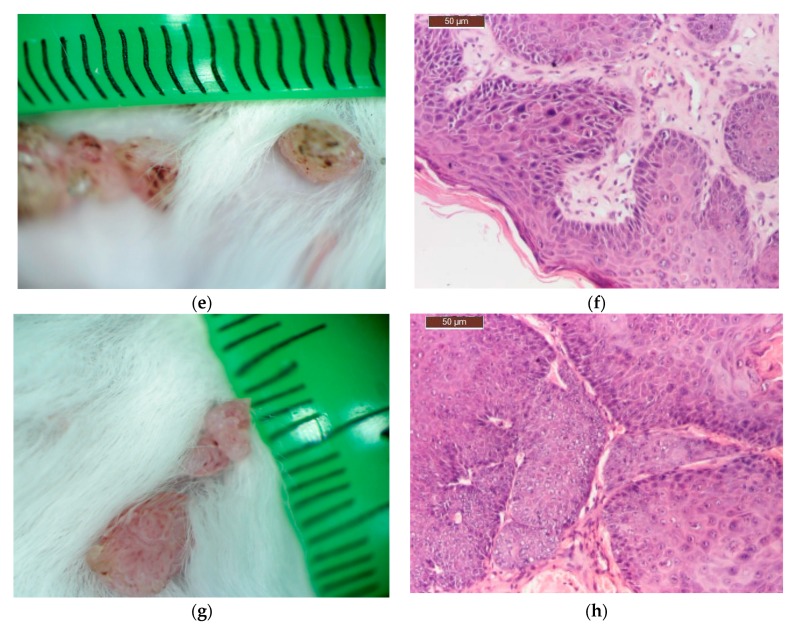
The tumour types chemically induced. The macroscopic tumour of origin and the H&E histological section pairs: (**a**,**b**) basal cell carcinoma (BCC); (**c**–**f**) squamous cell carcinoma (SCC); (**g**,**h**) basosquamous cell carcinoma (BSQCC).

The basic most predominant two types of skin cancer were induced: basal cell carcinoma (BCC) and squamous cell carcinoma (SCC). However, we also noticed one mouse with the rare methatypical form basosquamous cell carcinoma (BSQCC), as shown in Figure 5.

**Figure 6:**
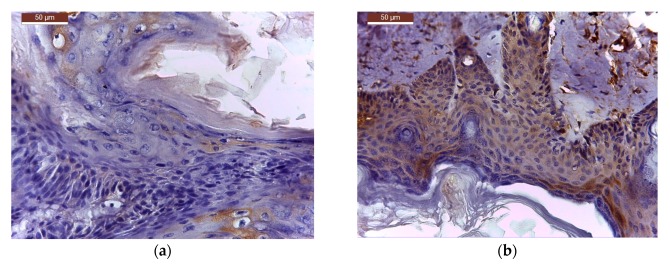

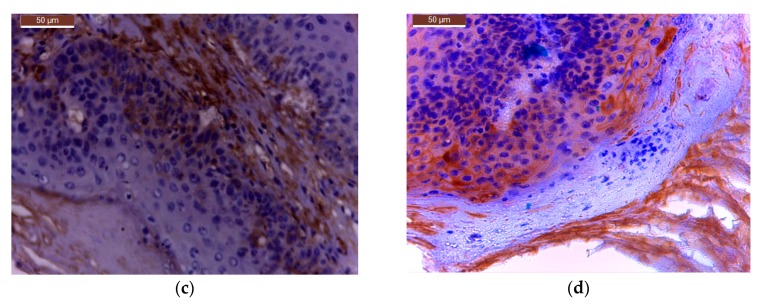
Bcl-2 expression in tumour sample treated with: (**a**) C_BCNU@HCLI; (**b**) cream BCNU; (**c**) base cream; (**d**) control saline.

**Figure 7:**
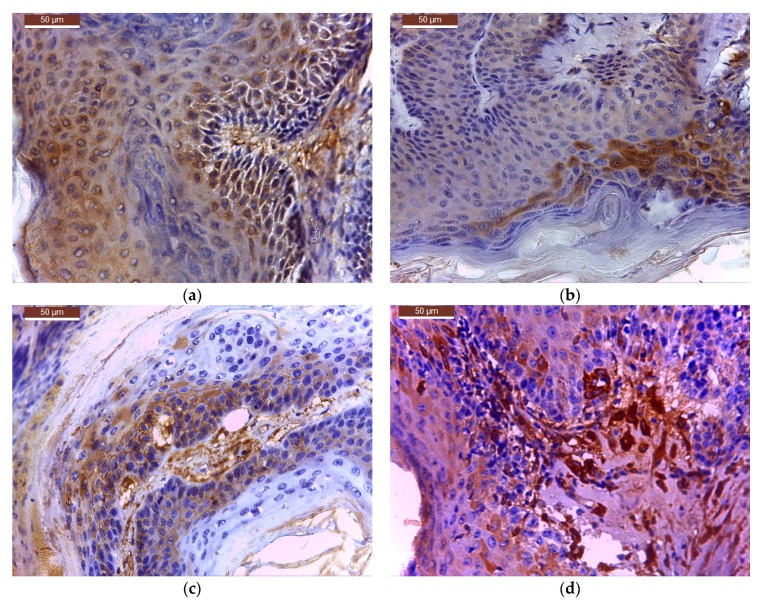
Cox-2 expression in tumour sample treated with: (**a**) C_BCNU@HCLI; (**b**) cream BCNU; (**c**) base cream; (**d**) control saline.

From six tumour-bearing mice (*n* = 6) of each treatment group, TIFFalyzer and IHC Profiler quantitative immunohistochemical analysis for the level of expression of bcl-2 (Figure 6, Table 4, Table 5 and Table 6) and cox-2 (Figure 7, Table 7, Table 8 and Table 9) was performed as described in methods. 

The treatment with C_BCNU@HCLI had a net effect on the expression of bcl-2, decreasing in a significant manner this anti-apoptotic factor in the tumour tissue by comparison to all the other types of treatments and thus reducing the survival chance of the tumour cells.

Based on this parameter, we believe that the new formulation deserves to be studied for further improvements.

Immunohistochemical sections for bcl-2 expression from all treated groups are presented in the Figure 6.

Quantitative levels of bcl-2 expression and inter-treatment comparisons for the tumour samples from all treated groups are presented in the following tables (Table 4, Table 5 and Table 6).

The treatment with C_BCNU@HCLI had also a net effect on the expression of cox-2, decreasing this tumour progression factor in a significant manner compared to all the other types of treatments and thus reducing the aggressiveness of the tumour.

Based on this second evaluation parameter, we believe that the new formulation deserves to be studied for further improvements.

Immunohistochemical sections for cox-2 expression from all treated groups are presented in the Figure 7.

Quantitative levels of cox-2 expression and inter-treatment comparisons for the tumour samples from all treated groups are presented in the following tables (Table 7, Table 8 and Table 9).

## 3. Discussion

We report about the qualities of a new modified delivery clinoptilolite-based system loaded with Carmustine used for local application in chemically-induced skin tumour in mice and evaluated for it’s in vivo efficacy, using as control parameters the level of expression of bcl-2 and cox-2 proteins, comparatively to the commercially formulation in use. The MDCS for Carmustine loading were prepared from the clinoptilolite tuff and subjected to several thermal and chemical treatments.

There are numerous studies that explored the use of zeolites in medicine for imaging mass spectrometry, wound treatment, and drug delivery [32,33,34,35,36]. Zeolites are used as drug delivery systems for small molecules due to their unique porous characteristics and when the pore size can be matched to the size of drug molecules [35]. Clinoptilolite was used either in its initial form or in modified form. Single or multiple step modifications of clinoptilolite by various chemical reagents gave specific properties that extended the field for medical use [10].

BCNU, a nitrosourea derivative, is an alkylating agent used as an anticancer drug that inhibits in a non-specific manner the cell-cycle phases by alkylating DNA and RNA. Carmustine proved efficacy for topical administration in early therapy of *Mycosis fungoides* in humans [11], after more than 30 years of use: complete response rates of approximately 63% to 75% in stage T1 patients, 21% in stage T3 patients, and up to 11% in stage T4 patients [11,37,38].

Porous parameters of our modified delivery clinoptilolite systems were evaluated using N_2_-sorption analysis to assess the porous features of the hosts and pore size distribution curves to assess the pore size homogeneity and sizes. These were in close agreement with previously published studies on bimodal porous feature of the clinoptilolite [9,10,39]. Typical surface areas and pore volumes were obtained for both of our monocationic NaCLI and HCLI matrices, but as expected, these values were lower for NaCLI matrix, which explained the lower loading degree of BCNU. 

The loading capacity of both matrices was about 5–6 mg BCNU/g of matrix. This was due to the dual porous feature of clinoptilolite matrix: the BCNU molecules can be accommodated in the channel-like micropores and the intraparticle mesopores [35].

The MDCS based on monocationic clinoptilolite behaved differently, both in terms of the amount of BCNU released and the release mechanism. The analysis of the release kinetics of BCNU from the MDCS revealed the fact that the clinoptilolite-based systems release BCNU in two different ways: BCNU@HCLI releases by Fick diffusion, as a consequence of Korsmeyer-Peppas model of data fitting, while BCNU@NaCLI releases the drug following a zero order kinetics. The results of curve fitting of in vitro BCNU release profile from MDCS confirmed that the zeolite matrix had a primordial role in defining the release kinetics of the active substance. The crystalline structure of clinoptilolite zeolite was the same, but the compensation cations differed in volume and hydration sphere, occupying different volumes in the pore system and inducing an electrostatic field of different intensity [40,41]. Therefore, BCNU release was strongly influenced by the compensation cations present in the zeolite channel system. Having these results in mind, it was easy to understand why BCNU@NaCLI system had demonstrated too slow release characteristics of BCNU for skin administration, but it might be suitable to be studied for the development of other pharmaceutical products with prolonged release and administration by other routes (e.g., oral, subcutaneous, or mucosal). The model for zero order kinetics describes the release of the drug from pharmaceutical matrix dosage forms with modified release, with a constant rhythm per unit of time independent of the concentration gradient [42,43]. BCNU release from BCNU@HCLI system by Fick diffusion recommends the use of this system for further study for the formulation of semisolid products meant for skin application [44,45].

We evaluated the release kinetics of BCNU from the BCNU@HCLI system dispersed in two semisolid formulations, cream type and gel type, obtained as it was presented in Methods. Better release of BCNU from the cream formulation compared to the gel formulation could be attributed to the strong lipophilic character of BCNU that had generated a greater affinity to the cream dispersion medium, thereby facilitating the diffusion and release process [46,47]. Based upon the results from in vitro dissolution of BCNU release study and measurement of permeation coefficient of BCNU, the C_BCNU@HCLI was selected for further testing in vivo in order to evaluate its effects.

All potentially new delivery systems of therapeutic agents should be evaluated using in vitro and in vivo models. In vitro evaluations give a lot of information, but in vivo experiments remain essential to characterize the new delivery systems. Therefore, we chose a widely used in vivo model of chemically induced skin cancer in mice [13] to test the effects of our new delivery system using as control parameters bcl-2 and on cox-2 protein expression levels. Deregulation of bcl-2 family members is involved in skin carcinogenesis [16,17]. Bcl-2, the first protein that was associated with translocations identified in follicular lymphoma, contributes to malignancy by inhibiting apoptosis [18]. The overexpression of cox-2 has been reported in pre-cancerous lesions, in several forms of cancer, including basal cell carcinoma or squamous cell carcinoma of the skin [15].

Calculating the mathematical “energy” of an image by determining the cumulative signal strength, or norm, of the digital file encoding that image can be used as the basis for accurate quantification of the amount of chromogen generated during immunohistochemistry [21]. The algorithm for QIHC provides the first mathematically valid approach for determining the absolute amount of chromogen present per pixel and shows that QIHC so performed can be used to accurately determine the amount of the target of interest in the tissues. IHC Profiler, integrates options for quantitative analysis of digital IHC images stained for either cytoplasmic or nuclear proteins. We have used both programs and obtained the same results for QIHC evaluation of the expression level of bcl-2 and cox-2 proteins. 

Local application of our new modified delivery clinoptilolite-based system loaded with Carmustine on the skin of laboratory animals with artificially chemically induced skin cancers, lead to decreased bcl-2 protein expression and cox-2 protein expression, so decreasing tumour survival chances and tumour aggressiveness, respectively, better than a reference Carmustine containing cream.

## 4. Materials and Methods 

### 4.1. Chemicals

Carmustine (98%) was purchased from Sigma Aldrich (Hamburg, Germany). Hydroxypropyl cellulose (HPC) 150–4000 cP was purchased from Nisso Chemical Europe GmbH (Düsseldorf, Germany); other chemicals (glycerol monostearate, cetil alcohol, petroleum jelly, propylene glycol, triethanolamine (TEA), ammonia solution (NH_3_) minimum 25%, hydrochloric acid 35–38% for analysis, oxalic acid dihydrate 99% for analysis, and sodium hydroxide dimethyl sulfoxide (NaOH DMSO)) were purchased from local sources. The reagents used for the high performance liquid chromatography (HPLC) analysis, Trifluoroacetic acid (TFA), acetonitrile (ACN) chromatographic purity, and glacial acetic acid, were purchased from Merck (Darmstadt, Germany); potassium phosphate, alcohol (pro analysis), and double-distilled water (Millipore, conductivity 0.01 μS/cm) were purchased from Sigma Aldrich (Hamburg, Germany). All reagents were used without purification. The deionized water was obtained from the Milli-Q water purification system, Elga Pure Lab (High Wycombe, UK). The initiator, 7,12-dimethylbenz[*a*]anthracene (DMBA), and the promoter, 12-*O*-tetradecanoylphorbol-13-acetate (TPA) were purchased from Ubichem (Redditch, UK). DMBA was solved in acetone to a concentration of 100 mg/100 mL. TPA was mixed in acetone to obtain a solution of 1%. Primary Antibody to cox-2, primary antibody to Bcl-2 Oncoprotein and secondary peroxidase anti-mouse IgG (H + L) were purchased from Vector Laboratories (Burlingame, CA, USA).

### 4.2. Preparation and Characterization of Carmustine-Loaded Clinoptilolite Modified Drug Delivery System

#### 4.2.1. Purification of Natural Clinoptilolite and Preparation of Monocationic Forms of Zeolite Matrix

Clinoptilolite was prepared in two ionic forms, HCLI and NaCLI, in order to immobilize the BCNU. The option for using these ionic forms was based on the amphoteric character of clinoptilolite, which can accept or release protons in an aqueous medium, mainly through an ion exchange process, thus being a good pH regulator. The two zeolite materials were prepared using volcanic tuff from the Mirsid quarry, Salaj county, Romania.

First, clinoptilolite tuff sample was subjected to grinding and sorting through screening. For this study, we have chosen clinoptilolite sample with particle size of ≈20 μm. Further, this sample was subjected to a mild thermal treatment to remove water, and then to several chemical treatments for purification: acid treatment with 5 N HCl at 25 °C, using the ratio solid:liquid = 1:10; secondary acid treatment with 2 N HCl solution concentration at the temperature of 90 °C, the ratio solid:liquid = 1:10; thermal treatment at 450 °C; treatment with 0.1 N solution of oxalic acid , the ratio solid:liquid = 1:10 at 25 °C followed by filtration, carefully washing and drying the filter. Monocationic H^+^ form was obtained by two-step ion exchange with NH_4_Cl with the ratio solid:liquid of 1:10 exchange 1M solution. After the ion exchange, the sample was calcined for 6 h at 450 °C. Monocationic Na^+^ form was obtained by ion exchange in two stages using NaCl. The samples prepared in this way were stored in a desiccator and protected from moisture. The samples prepared in this way were used to prepare MDCS for loading carmustine.

#### 4.2.2. Drug Loading and Obtain of the Modified Delivery Clinoptilolite–Based Systems 

BCNU is practically insoluble in water but soluble in ethanol and DMSO and highly soluble in lipids. Therefore, an alcoholic solution was chosen to perform BCNU immobilization tests. The amount of BCNU immobilized on the two zeolite matrices was determined on the basis of its disappearance from the solution by high performance liquid chromatography. Practically an amount of 1 g of each of the two zeolite matrix forms, HCLI and NaCLI, respectively, were mixed with 300 mL of 1 mg/mL of BCNU alcoholic solution. Samples were stored with magnetic stirring at 60 rpm, at room temperature for 3 h. 

After separation by centrifugation and washing, the samples were dried at room temperature in the dark and they were labelled as BCNU@HCLI and BCNU@NaCLI, respectively. 

#### 4.2.3. HPLC Assay of BCNU 

The chromatographic method, applied for the assessment of the loading capacity of MDCS with BCNU and for the in vitro dissolution tests, was developed and validated in-house. 

The amount of retained BCNU was determined using the HPLC method, developed, validated, verified and revalidated in house with a HPLC Dionex Ultimate^TM^ 3000 system (Thermo Fisher Scientific Inc., Cleveland, OH, USA) equipped with a UV-VIS Diode Array Detector. The working conditions optimized for the chromatographic system involved the utilization of the mobile phase consisting in a 0.1% TFA in H_2_O solution (solvent A) and acetonitril (solvent B), in a ratio of 60:40 (*v*/*v*), working temperature 30 °C in column Hypersil GOLD (Thermo Fisher Sciencific Inc., Cleveland, OH, USA), 4.6 × 150 mm, 5 μm, injection of a sample volume of 10 μL and chromatogram recording with detection at a wavelength of 230 nm. The method was validated by measuring the following parameters: method repeatability and intermediate precision for a concentration of 0.1 mg/mL, when we obtained relative standard deviation (RSD) values ≤2 for peak area, retention time and concentration; method linearity in the concentration range of 0.25–2 mg/mL, equation of curve calibration *y* = 225.19*x* + 0.1074) determination coefficient (*R*^2^) = 0.9995); detection limit—0.00944 mg/mL and quantification limit—0.0286 mg/mL. 

#### 4.2.4. Characterization Methods of the Modified Delivery Clinoptilolite-Based Systems

##### The Assessment of Porous Parameters of Modified Delivery Clinoptilolite-Based Systems 

The nitrogen sorption isotherms of MDCS, before and after drug loading process, were recorded on a Quantachrome Nova 2200 Instrument and Pore Size Surface Area Analyzer (Beckman Coulter, Inc., Atlanta, GA, USA) at −196 °C. Before measurements, the samples were outgassed under high vacuum at room temperature for 12 h. The Brunauer–Emmett–Teller (BET) specific surface area (S_BET_ (m^2^/g)) was calculated from the linear part of the BET plot. Micropore area (S_μ_ (m^2^/g)) was determined by t-plot method. The average pore diameter (Dp (nm)) was estimated using the desorption branch of the isotherm and the BJH method. The volume of liquid nitrogen adsorbed P/P_0_ = 0.95 was used to assess the total pore volume (TPV (cm^3^/g)).

##### In Vitro Drug Release Profile of BCNU from Modified Delivery Clinoptilolite-Based Systems

The in vitro dissolution tests of BCNU from the BCNU@HCLI and BCNU@NaCLI systems were performed using as a dissolution media a mixture of ethyl alcohol: phosphate-buffered saline (EtOH:PBS) in a 3:1 ratio. The test was performed on a SR8 Plus Series (AB & L JASCO, Chatsworth, CA, USA) apparatus 2 (paddles) equipped with a Dissoette autosampler (Hanson Research Corporation, Chatsworth, CA, USA) according to the following protocol: a specific amount of MDCS containing 25 mg of BCNU was introduced into containers having 50 mL of dissolution media, with a bath temperature of 37 ± 0.5 °C, rotation speed 50 rpm; the sampling interval was set at every 60 min during 12 h. Aliquots (1 mL) were withdrawn and subjected to high-performance liquid chromatography coupled to diode array detector (HPLC-DAD) method described above, in order to determine the amount of BCNU released. After every sampling, the aliquots were replaced with equal volumes of medium at 37 °C. 

In order to highlight the characteristics of systems with modified release for the matrices under investigation, in the in vitro dissolution test, we also recorded the dissolution profile of the unincorporated BCNU.

##### Analysis of In Vitro BCNU Release Kinetics 

In order to predict and correlate the behaviour of the in vitro BCNU release from the MDCS systems, a suitable mathematical model was used. Thus, the experimental data obtained from the in vitro dissolution tests were investigated using four predictable models: zero-order and first-order kinetics, Higuchi and Korsmeyer-Peppas models [48]. 

Let *C*_0_ be the initial concentration of drug in the pharmaceutical dosage form and *C_t_* the concentration of drug in the pharmaceutical dosage form at time *t*. If *F_t_* = 1 − *C_t_*/*C*_0_ denotes the released percentage at time *t*, then the release formulae corresponding to the four models are

*F_t_* = *K*_0_ × *t* (the zero-order model), (1)

*F_t_* = 1 − *e*^−*K*_1_×*t*^ (the first-order model), (2)

$$F_{t}=K_{H}\times \sqrt{t}\;(\mathrm{the}\;\mathrm{Higuchi}\;\mathrm{model})$$ (3)

(4) *F_t_* = *K_P_* × *t^n^* (the Korsmeyer-Peppas model)

Here, *n* is the release exponent corresponding to the Korsmeyer-Peppas model, while *K*_0_, *K*_1_, *K_H_*, and *K_P_* are the release rate constants corresponding to the zero-order, the first-order, the Higuchi, and the Korsmeyer-Peppas model, respectively. These constants have been fitted using linear and nonlinear regression in Matlab 7.1 and the results were displayed in Table 2. The determination coefficient was the criteria for selecting the model that most dependably describes the release profile of each formula. In a reliable prediction model, the value of *R*^2^ is as close to 1 as possible [49,50].

#### 4.2.5. Development of Semisolid Dosage form with BCNU@HCLI System

##### Preparation of Semisolid Dosage Forms 

Two types of semisolid dosage forms were formulated by dispersing the BCNU@HCLI system, which exhibited the best dissolution characteristics in the dissolution in vitro test, in a HPC gel and in a cream base according to the data presented in Table 10. The dispersed BCNU@HCLI quantity was calculated according to the matrix loading degree so that the semisolid products had a final 0.4% BCNU concentration.

##### In Vitro Dissolution BCNU Release Study and Measurement of the Permeation Coefficient of BCNU

The in vitro dissolution test was performed on the Enhancer cell having a diameter of 2.5 cm, employing the SR 8 Plus Series device (AB & L Jasco, Cremella, Italy), according to the following protocol: dissolution media: a mixture of ethyl alcohol : EtOH:PBS in a 3:1 ratio, 100 mL; mass of sample: for each formulation studied, 1 g; two types of synthetic membrane (supplied by Millipore, Merck, Darmstadt, Germany): Nylon membrane with a pore diameter Ø = 45 μm and collagen membrane (Strat-M^®^ membrane, Sigma Aldrich, Hamburg, Germany); temperature: 37 °C ± 0.2 °C; sampling interval: the test was carried out over a period of 12 h, every 60 min we drawn out a sample volume of 1 mL, which was replaced with fresh medium; speed: 100 rpm. The synthetic membrane was placed in the dissolution medium for 24 h before the in vitro test.

The in vitro permeability coefficient of BCNU was calculated by the following equation:

(5) $$K_{p}=\frac{J}{C}\times A,$$

where: *K_P_*—coefficient of permeability (cm/h); *J*—rate of drug substance permeation or stationary phase flow (μg/h); *C*—concentration in the donor compartment (μg/mL); *A*—contact surface area (cm^2^).

### 4.3. In Vivo Evaluation of the Modified Delivery Clinoptilolite-Based Systems Loaded with Carmustine, Using as Control Parameters the Level of Expression of bcl-2 and cox-2 Proteins after the Local Application on the Skin Tumour

#### 4.3.1. Animals

The protocol of the experimental study was approved (17 September 2015, PN-II-PT-PCCA- 2013-4-2024) by the Institutional Ethical Committee of the University of Medicine and Pharmacy “Gr. T. Popa”. Animals were male CD1 Swiss mice (seven weeks old, weighing 18 ± 2 g), purchased from “Cantacuzino” Institute Bucharest, Romania. Animal care and handling were done according to the international guidelines. The animals were housed in polypropylene cages with controlled environmental conditions of temperature (21 ± 2 °C), humidity (50–70%), and light (12-h light/dark cycle) and had ad libitum access to food and water.

#### 4.3.2. Experimental Design

A two-step modified protocol (DMBA followed by TPA) was used to induce skin tumours in mice [13,51,52,53]. The dorsal region of the mice was shaved with an electric clipper. Two days later, mice were treated topically each time with 200 nmol DMBA in 200 μL acetone, with three administrations in two-week at three-day interval, and control mice received 200 μL acetone. After two weeks, the tumour initiation by DMBA was promoted by the topical application each time with 200 μL TPA solution 1% in acetone, three times per week, for 16 weeks. 

After 16 weeks of carcinogen treatment, the animals were divided into groups of 12 animals/group and the substances were applied on the skin once a day, daily, for three weeks, as follows:

- Group I (experimental: C_BCNU@HCLI): topical application of 0.5 g Carmustine in the new delivery system/mouse;
- Group II (control for Carmustine: Cream BCNU): topical application of 0.5 g Carmustine^®^ cream/mouse);
- Group III (control for the base: Base cream): topical application of 0.5 g base cream/mouse);
- Group IV (Saline control): topical application of 100 μL NaCl 9‰/mouse.

#### 4.3.3. Quantitative Immunohistochemistry Analysis for the Evaluation of the Expression of bcl-2 and cox-2 Proteins, Using Computer Aided Diagnosis Software: TIFFalyzer and IHC Profiler

On the completion of the third week of local application of different treatments, skin and tumour tissue samples from six tumour-bearing mice from each type of treatment, were collected for histopathological examination. Skin and tumour tissue samples were fixed with Bouin, dehydrated with ethanol and embedded in paraffin. After cutting, 4 μm sections were dewaxed and blocked epitopes in the sections were revealed by heating at 95 °C in 10 mmol citrate acid buffer pH6 for 10 min in a microwave oven and then were left at room temperature for 20 min. The slides were then washed twice in PBS (pH = 7.5) for 5 min and incubated with the primary antibody—purified Mouse Anti human bcl-2 or Mouse Anti human cox-2 antibody, diluted 1:100, at room temperature in humid chamber overnight. After washing with PBS, slides were then incubated with the secondary antibody, horseradish peroxidase (HRP) Goat anti Mouse IgG for 1 h in humid chamber at 4 °C, then washed with PBS and incubated with the DAB (3,39-diaminobenzidine tetrahydrochloride) substrate for 5 min and counter-stained with Harris haematoxylin, clarified in xylene. Slides were mounted and examined with a Leica DM 750 microscope (model Leica DM 750 Microsystems Ltd., Heerbrugg, Switzerland). For all cross sections, controls were obtained from consecutive sections by using the same procedure, except that they were not exposed to primary antibody. 

##### TIFFalyzer

This is an algorithm for performing QIHC. Chromogen abundance was quantified by quantitative immunohistochemistry as previously described by Matkowskyj et al. [21,23,24]. This technique relies on calculating the cumulative signal strength, or mathematical energy (EM) [54] of the image under consideration. The amount of chromogen per pixel was determined by subtracting the value for chromogen abundance of the control slide from that in the homologous region of the experimental slide. Data relative to the amount of total chromogen present was given as energy units per pixel (EU/pixel). For all images, the relevant stained region was isolated from the rest of the image using Adobe Photoshop version 18 (Adobe Systems, San Jose, CA, USA) and used for chromogen quantification using the MATLAB program running TIFFalyzer code (developed by Randal Cox, Bioinformatics Group, Departments of Genetics and Medicine, University of Illinois, Chicago, IL, USA and modified for batch analysis by Iulian Stoleriu, Faculty of Mathematics, “Al. I. Cuza” University, Iasi, Romania). 

##### IHC Profiler

A simple method of automated digital IHC image analysis algorithm for an unbiased, quantitative assessment of antibody staining intensity in tissue sections was used for the quantification of the expression of Bcl-2 and cox-2 proteins. This software is an open source plugin named IHC Profiler [55], which is compatible with the ImageJ software and is a demonstrated method for IHC analysis using colour deconvolution and computerized pixel profiling leading to the assignment of an automated score to the respective image. The result of colour deconvolution leads to the production of three images, namely, DAB, haematoxylin and a complimentary image. For analyzing the cytoplasmic staining pattern, a histogram profile is assigned for the deconvoluted DAB image, which is a plot between the intensity values of the pixels (*X* axis) vs. the number of pixels representing the intensity (*Y* axis). In digital image analysis, the pixel intensity values for any colour range from 0 to 255, wherein, 0 represents the darkest shade of the colour and 255 represent the lightest shade of the colour as standard. Keeping in view the standard grading procedure, the histogram profile was divided into four zones: high positive, positive, low positive, and negative. These four zones were equally divided on the pixel colour intensity bar. The intensity range for the positive zone was from 61 to 120, 121 to 180 for the low positive zone, and 181 to 235 for the negative zone, respectively. It was determined that the pixels with intensity values ranging from 235–255 predominantly represent fatty tissues which are occasionally present but do not typically contribute to pathological scoring and were therefore excluded from the score determination zones. A simple algebraic formula was conceptualized for score assignment to the IHC images.

$$\mathrm{Score}=\frac{\left(\mathrm{Number}\;\mathrm{of}\;\mathrm{pixels}\;\mathrm{in}\;a\;\mathrm{zone}\right)\times \left(\mathrm{Score}\;\mathrm{of}\;\mathrm{the}\;\mathrm{zone}\right)}{\mathrm{Total}\;\mathrm{number}\;\mathrm{of}\;\mathrm{pixels}\;\mathrm{in}\;\mathrm{the}\;\mathrm{image}}$$ (6)

Wherein the score of the zone is assigned as 4 for the high positive zone, 3 for the positive zone, 2 for the low positive zone, and 1 for the negative zone. The global score is the sum of the partial scores for the four zones. The higher the score, the higher is the level of expression in the sample. 

#### 4.3.4. Statistical Analysis

Data were expressed as mean standard deviation. Statistical analysis was performed using IBM SPSS Statistics version 20.0 (SPSS, Inc., Chicago, IL, USA), to evaluate the difference between the means of the scores of each zone: high positive, positive, low positive, and negative. The significance of difference was obtained by performing the two-tailed t Student test for independent samples (unequal variances) and confidence interval set at 95%. Values of p lesser than 0.05 were considered significant.

## 5. Conclusions

We found that local application of our new modified delivery clinoptilolite-based system loaded with Carmustine on the skin of laboratory animals with artificially chemically induced skin cancers lead to decreased bcl-2 protein expression and cox-2 protein expression, so decreasing tumour survival chances and tumour aggressiveness, respectively, better than a reference Carmustine containing cream. 

Our new formulation, a Carmustine-loaded clinoptilolite delivery cream, could be a better and more effective drug delivery tool for the topical system.

## Figures and Tables

**Table 1:** Porous properties and drug content of modified delivery clinoptilolite-based systems.

Clinoptilolite Matrices	HCLI	NaCLI	BCNU@HCLI	BCNU@NaCLI
BET specific surface area (S_BET_-m^2^/g)	104 ± 3.28	58 ± 1.51	93.76 ± 1.68	53.94 ± 2.11
Total pore volume (cm^3^/g)	0.122 ± 0.011	0.068 ± 0.002	0.1101 ± 0.006	0.063 ± 0.001
Micropore area (m^2^/g)	44 ± 0.89	18.5 ± 0.09	39.66 ± 0.31	17.20 ± 0.67
Pore diameter (nm)	3.70 ± 0.03	3.78 ± 0.01	3.36 ± 0.01	3.51 ± 0.02
Loaded drug (mg BCNU/g matrix)	-	-	5.9717 ± 0.09	5.2239 ± 0.12

Data are presented as mean ± Standard deviation (SD). S_BET_: Brunauer–Emmett–Teller (BET) specific surface area; BCNU@HCLI: clinoptilolite hydrogen form loaded with carmustine; BCNU@NaCLI: clinoptilolite sodium form with carmustine.

**Table 2:** Results of curve fitting of in vitro BCNU release profile from modified delivery clinoptilolite-based systems.

Kinetic Model	Model Coefficients	Modified Delivery Clinoptilolite-Based System	
		BCNU@HCLI	BCNU@NaCLI
Zero-order	*K*_0_	0.246	0.166
	*R*^2^	0.852	0.993
First-order	*K*_1_	0.003	0.002
	*R*^2^	0.899	0.990
Higuchi	*K_H_*	2.300	1.500
	*R*^2^	0.969	0.856
Korsmeyer-Peppas	*n*	0.650	0.850
	*K_P_*	1.192	0.208
	*R*^2^	0.974	0.971

*n* is the release exponent corresponding to the Korsmeyer-Peppas model; *K*_0_, *K*_1_, *K_H_* and *K_P_* are the release rate constants corresponding to the zero-order, the first-order, the Higuchi, and the Korsmeyer-Peppas model; *R*^2^ is determination coefficient.

**Table 3:** Parameters specific to BCNU permeation in the semisolid dosage forms (*n* = 3).

Sample Formula	Nylon Membrane		Collagen Membrane	
	*J_ss_* (μg/cm^2^/h)	*K_P_* × 10^−6^ (cm/h)	*J_ss_* (μg/cm^2^/h)	*K_P_* × 10^−6^ (cm/h)
**G_BCNU@HCLI**	7.143 ± 1.924	86.111	7.156 ± 3.904	88.221
**C_BCNU@HCLI**	7.067 ± 1.790	88.034	7.741 ± 2.310	98.078

Data are presented as mean ± SD. *K_P_*: coefficient of permeability; *J_ss_*: steady state flux.

**Table 4:** Bcl-2 expression level: quantitative intensity of the chromogen (diaminobenzidine, DAB) expressed as EU/pixel (Energy Units/pixel) of the new formulation and the control type treatments; (TIFFalyzer); standard error (SE).

DAB (EU/Pixel)	C_BCNU@HCLI	Cream BCNU	Base Cream	Control Saline
Mean	75.63	106.33	185.66	157
SD	±23.59	±15.12	±59.33	±45.15
SE	8.91	5.71	22.42	17.06

**Table 5:** Bcl-2 expression level: the difference between the means obtained by performing the two-tailed *t*-Student test for independent samples (unequal variances) and confidence interval set at 95%; (TIFFalyzer).

TREATMENT	C_BCNU@HCLI	Cream BCNU	Base Cream	Control Saline
C_BCNU@HCLi	NS	*p* < 0.05	*p* < 0.001	*p* < 0.01
Cream BCNU	*p* < 0.05	NS	*p* < 0.01	*p* < 0.05
Base Cream	*p* < 0.001	*p* < 0.01	NS	NS
Control Saline	*p* < 0.01	*p* < 0.05	NS	NS

**Table 6:** Bcl-2 expression level: scoring and levels of significance in paired comparison of the new formulation and the control type treatments; (IHC Profiler).

	C_BCNU@HCLI	Cream BCNU	Base Cream	Control Saline
SCORING	1.576	2.434	2.926	2.736
High Positive	-	*p* < 0.05	*p* < 0.05	*p* < 0.05
Positive	-	*p* < 0.05	*p* < 0.05	*p* < 0.05
Low positive	-	NS	*p* < 0.05	NS
Negative	-	*p* < 0.05	NS	NS

**Table 7:** Cox-2 expression level: quantitative intensity of the chromogen (DAB) expressed as EU/pixel of the new formulation and the control type treatments; (TIFFalyzer).

DAB (EU/Pixel)	C_BCNU@HCLI	Cream BCNU	Base Cream	Control Saline
Mean	67.41	106.86	159.46	223
SD	±18.70	±20.63	±54.26	±80.23
SE	7.07	7.80	20.51	30.32

**Table 8:** Cox-2 expression level: the difference between the means obtained by performing the two-tailed *t*-Student test for independent samples (unequal variance) and confidence interval set at 95%; (TIFFalyzer).

	C_BCNU@HCLI	Cream BCNU	Base Cream	Control Saline
C_BCNU@HCLI	NS	*p* < 0.01	*p* < 0.01	*p* < 0.05
Cream BCNU	*p* < 0.01	NS	*p* < 0.05	*p* < 0.01
Base Cream	*p* < 0.01	*p* < 0.05	NS	NS
Control Saline	*p* < 0.05	*p* < 0.01	NS	NS

**Table 9:** Cox-2 expression level: scoring and levels of significance in paired comparison of the new formulation and the control type treatments; (IHC Profiler).

	C_BCNU@HCLI	Cream Bcnu	Base Cream	Control Saline
SCORING	1.949	2.726	2.732	3.229
High Positive	-	*p* < 0.05	*p* < 0.05	*p* < 0.05
Positive	-	*p* < 0.05	*p* < 0.05	*p* < 0.05
Low positive	-	*p* < 0.05	*p* < 0.05	NS
Negative	-	*p* < 0.05	*p* < 0.05	*p* < 0.05

**Table 10:** Formulations of semisolid dosage forms with BCNU@HCLI: composition and abbreviation.

Raw Materials	Sample Formulation	
	G_BCNU@HCLI	C_BCNU@HCLI
HPC	3.5 g	-
Glycerol monostearate	-	12 g
Cetil alcohool	-	1 g
Propylene glycol	3 g	3 g
Triethanolamine	1 g	-
Petroleum jelly	-	12 g
BCNU@HCLI	6.40 g	6.40 g
Purified water	... up to 100 g ...	... up to 100 g ...

HPC: hydroxypropyl cellulose.
